# Characterization of cervico-vaginal microbiota in women developing persistent high-risk Human Papillomavirus infection

**DOI:** 10.1038/s41598-017-09842-6

**Published:** 2017-08-31

**Authors:** Monica Di Paola, Cristina Sani, Ann Maria Clemente, Anna Iossa, Eloisa Perissi, Giuseppe Castronovo, Michele Tanturli, Damariz Rivero, Federico Cozzolino, Duccio Cavalieri, Francesca Carozzi, Carlotta De Filippo, Maria Gabriella Torcia

**Affiliations:** 1 Department of Neuroscience, Psychology, Drug Research and Child Health (NEUROFARBA), University of Florence, Meyer Children Hospital, Florence, Italy; 2 S.C. Screening e Prevenzione Secondaria, Istituto per lo Studio e la Prevenzione Oncologica (ISPO), Florence, Italy; 3 Department of Experimental and Clinical Medicine, University of Florence, Florence, Italy; 4 Laboratorio Regionale HPV e Biologia Molecolare, LRPO, Istituto per lo Studio e la Prevenzione Oncologica (ISPO), Florence, Italy; 5 Department of Biology, University of Florence, Florence, Italy; 6 Department of Experimental and Clinical Biomedical Sciences, University of Florence, Florence, Italy; 7 Institute of Biology and Agrarian Biotechnology (IBBA), National Research Council (CNR), Pisa, Italy

## Abstract

Changes in cervico-vaginal microbiota with *Lactobacillus* depletion and increased microbial diversity facilitate human papillomavirus (HPV) infection and might be involved in viral persistence and cancer development. To define the microbial Community State Types (CSTs) associated with high-risk HPV−persistence, we analysed 55 cervico-vaginal samples from HPV positive (HPV+) women out of 1029 screened women and performed pyrosequencing of 16S rDNA. A total of 17 samples from age-matched HPV negative (HPV−) women were used as control. Clearance or Persistence groups were defined by recalling women after one year for HPV screening and genotyping. A CST IV subgroup, with bacterial genera such as *Gardnerella*, *Prevotella*, *Megasphoera*, *Atopobium*, frequently associated with anaerobic consortium in bacterial vaginosis (BV), was present at baseline sampling in 43% of women in Persistence group, and only in 7.4% of women in Clearance group. *Atopobium* genus was significantly enriched in Persistence group compared to the other groups. Sialidase-encoding gene from *Gardnerella vaginalis*, involved in biofilm formation, was significantly more represented in Persistence group compared to the other groups. Based on these data, we consider the CST IV-BV as a risk factor for HPV persistence and we propose *Atopobium spp* and sialidase gene from *G. vaginalis* as microbial markers of HPV−persistence.

## Introduction

Cervical cancer (CC) is one of the most common cancer in women, with an estimated incidence of 485 000 new cases and 236 000 deaths in 2013^1^, causing 6.9 million disability-adjusted life-years (DALYs). Persistence of oncogenic human papillomavirus (HPV) infection contribute to the development of CC.

While the virus is cleared in more than 90% of infections within 6–18 months^2–4^, viral persistence occurs in almost 10% of infected women. The factors responsible of persistence, as well those that promote and initiate the carcinogenesis process, need to be fully elucidated. Many other factors such as immunodeficiency, age, smoking, oral contraceptives and *Chlamydia trachomatis* infection are related with higher persistence rates^5, 6^. Recently, several scientific reports indicated the role of vaginal microbiota in the acquisition and persistence of HPV and risk of CC development^7^.

In the majority of human body sites, highly diverse microbial communities are generally considered a signature of health^8, 9^. However, in the vaginal environment, health is commonly associated with low microbial diversity and prevalence of only a few species of *Lactobacillus* ^10–13^. *Lactobacillus* spp. prevent colonization of exogenous pathogens by producing lactic acid, bacteriocins and reactive oxygen species (ROS), and compete with them for adherence sites to mucous layer^13–15^. Five major community-state types (CSTs) discriminate vaginal microbiota in healthy women^11^. *Lactobacillus crispatus*, *L. gasseri*, *L. iners* and *L. jensenii* dominate CST I, II, III and V respectively, while depletion of *Lactobacilli* identifies CST IV^11^. In previous studies differences in vaginal microbiota by ethnicity was observed^16^. African American women may have increased *L. iners* and decreased *L. crispatus* compared with Caucasian women^16, 17^.

Anaerobic bacterial species of *Gardnerella*, *Prevotella*, *Peptostreptococcus* genera and/or aerobic bacteria of *Enterobacteriacee* family usually populate vaginal environment depleted of *Lactobacillus* species^18–20^.

CST IV is frequently associated with bacterial vaginosis (BV), the most common vaginal infection in women of reproductive age^7^. In this disorder *Atopobium vaginae*, Clostridiales and selected *Gardnerella vaginalis* strains usually form biofilm on the vaginal epithelium, resistant to antibiotic therapies^21^.

BV emerged as a public health problem due to its association with sexual transmitted infections including human immunodeficiency virus (HIV) and HPV^22–26^. CST IV is also frequent in aerobic vaginitis (AV). In this disorder, *Lactobacillus* spp. are predominantly replaced with enteric commensals or pathogens. Group B *Streptococci* (GBS), *Escherichia coli*, and *Staphylococcus aureus* are microorganisms most frequently associated with AV^27–30^.

In this paper, to define the Community State Types (CSTs) associated with HPV-persistence, we used cervico-vaginal samples collected in a study for HPV screening program. Cervico-vaginal microbiota was characterized by metagenomic analysis in samples of HPV positive women and in HPV negative, selected as control. Microbial profiles were associated with viral clearance or persistence.

## Results

### Study population

Cervico-vaginal samples were collected in the contest of a pilot study aimed to evaluate the efficacy of HPV test in a primary screening program involving 1029 women aged between 26–64 years old (see Methods).

A total of 55/1029 samples were positive for high risk HPV (HR-HPV+) at baseline screening. We were able to determine the HPV genotype in 50/55 samples collected at baseline screening and no significant prevalence of HPV genotype was recorded. Multiple HPV genotype were present in 13/50 samples (Supplementary Table S1).

All HR-HPV+ women attended the one-year follow up visit and were subjected to a second HR-HPV assay. The results of the second assay were used to stratify the baseline sampling in (i) Clearance Group (n = 27), HR-HPV+ women in which infection clears and had no evidence of HR-HPV DNA after one year; (ii) Persistence group (n = 28), HR-HPV+ women who developed persistent infection and maintained the expression of at least one of HPV-DNA genotypes revealed at baseline sampling. HR-HPV+ women were triaged to cytological testing. When Atypical Squamous Cells of Undetermined Significance (ASC-US) or a more severe lesion was ascertained, women were referred for colposcopy to define the grade (low or high) of lesions (see Methods). We reported Cervical Intraepithelial Neoplasia grade 1 (CIN1) and/or condiloma in 6 out of 28 (21.4%) and CIN grade 2 (CIN2) in 2 out of 28 (7.14%), as shown in Supplementary Table S1.

A total of 17 samples from HPV negative (HPV−) women matched for age were selected as control group. All the available epidemiologic and clinical information about the enrolled women were reported in Table 1, as well as the HPV genotyping and the results of colposcopy at one-year follow up in Supplementary Table S1.

**Table 1:** Summary of the characteristics available for the women in the three group (Clearance, Persistence, and Control). For age range, mean and standard error are indicated. With n (%), number of women and percentage are reported.

Characteristics	HPV+ Clearance group (n = 27)	HPV+ Persistence group (n = 28)	HPV−Control group(n = 17)
Ethnicity	Caucasian	Caucasian	Caucasian
Age range (mean ± se)	26–64 (45 ± 11)	26–56 (41 ± 9)	27–57 (43 ± 9)
Postmenopausal, n (%)	7 (25.9%)	4 (14.8%)	4 (23.5%)
Available HPV genotyping, n (%)	23 (85.2%)	27 (96.4%)	0 (0%)
Colposcopy report (after one year), n (%)	0 (0%)	CIN1 6 (21.4%)	0 (0%)
		CIN2 2 (7.14%)	
		CIN3 1 (3.5%)	

### Cervico-vaginal microbiota characterization

Meta-taxonomic investigation of cervico-vaginal microbiota was performed *via* pyrosequencing of the V3-V5 hypervariable region of 16S rDNA gene, amplified in DNA from samples of HPV+ and HPV−women, as controls. In Supplementary Fig. S1A–C and Table S2A–D, we reported taxonomic profiles at phylum, family, genus level in all enrolled women.

As expected, *Lactobacillus* represented the most abundant genus of cervico-vaginal microbiota in the three groups (Supplementary Table S2E). However, differences in the distribution of *Lactobacillus* species were found (Table 2). *L. crispatus* was the most abundant *Lactobacillus* species in the Control and in Clearance group (Table 2 and Supplementary Table S2E).

**Table 2:** Average of relative abundances of the 20 prevalent genera in cervico-vaginal microbiota in the three groups. *Indicated the most abundant species of *Lactobacillus* were reported.

OTU	HPV+ Clearance group	HPV+ Persistence group	HPV− Control group
Firmicutes|*Lactobacillus*	53.1%	50.3%	57.3%
Firmicutes|Lactobacillus|crispatus*	28.5%	16.7%	29.9%
Firmicutes|Lactobacillus|gasseri*	7.78%	3.54%	3.32%
Firmicutes|Lactobacillus|iners*	16.5%	29.8%	23.7%
Firmicutes|Lactobacillus|jensenii*	0.01%	0.04%	0.01%
Actinobacteria|*Gardnerella*	6.2%	18.9%	12.5%
Actinobacteria|*Bifidobacterium*	9.2%	3.0%	10.8%
Bacteroidetes|*Prevotella*	3.0%	6.1%	4.5%
Actinobacteria|*Atopobium*	1.3%	5.5%	1.7%
Proteobacteria|*Escherichia/Shigella*	7.6%	0.2%	0.1%
Firmicutes|*Streptococcus*	1.9%	1.2%	1.2%
Proteobacteria|*Gluconacetobacter*	1.3%	1.3%	1.1%
Proteobacteria|*Sphingomonas*	1.2%	1.3%	1.0%
Actinobacteria|*Propionibacterium*	1.7%	0.6%	1.5%
Fusobacteria|*Sneathia*	0.3%	2.2%	0.0%
Firmicutes|*Pediococcus*	0.9%	1.1%	0.8%
Firmicutes|*Megasphaera*	1.2%	1.1%	0.0%
Cyanobacteria|*Streptophyta*	0.8%	0.8%	0.8%
Actinobacteria|*Brevibacterium*	1.2%	0.5%	0.0%
Proteobacteria|*Pseudomonas*	0.8%	0.4%	0.3%
Actinobacteria|*Corynebacterium*	0.4%	0.5%	0.3%
Proteobacteria|*Gluconobacter*	0.3%	0.3%	0.3%
Firmicutes|*Aerococcus*	0.1%	0.0%	1.1%
Firmicutes|*Enterococcus*	0.6%	0.1%	0.0%

When a depletion in *Lactobacillus* spp. (<60% of relative abundance for sample) was observed, enrichment in aerobic and anaerobic bacteria, likely derived from gut environment or faecal contamination, was found in the three groups. In particular, bacterial genera such as *Gardnerella*, *Prevotella*, *Atopobium*, *Escherichia/Shigella* and *Streptococcus*,associated with bacterial vaginosis^11, 22, 23, 31^, were frequently found in our cohorts (Table 2).

**Figure 1:**
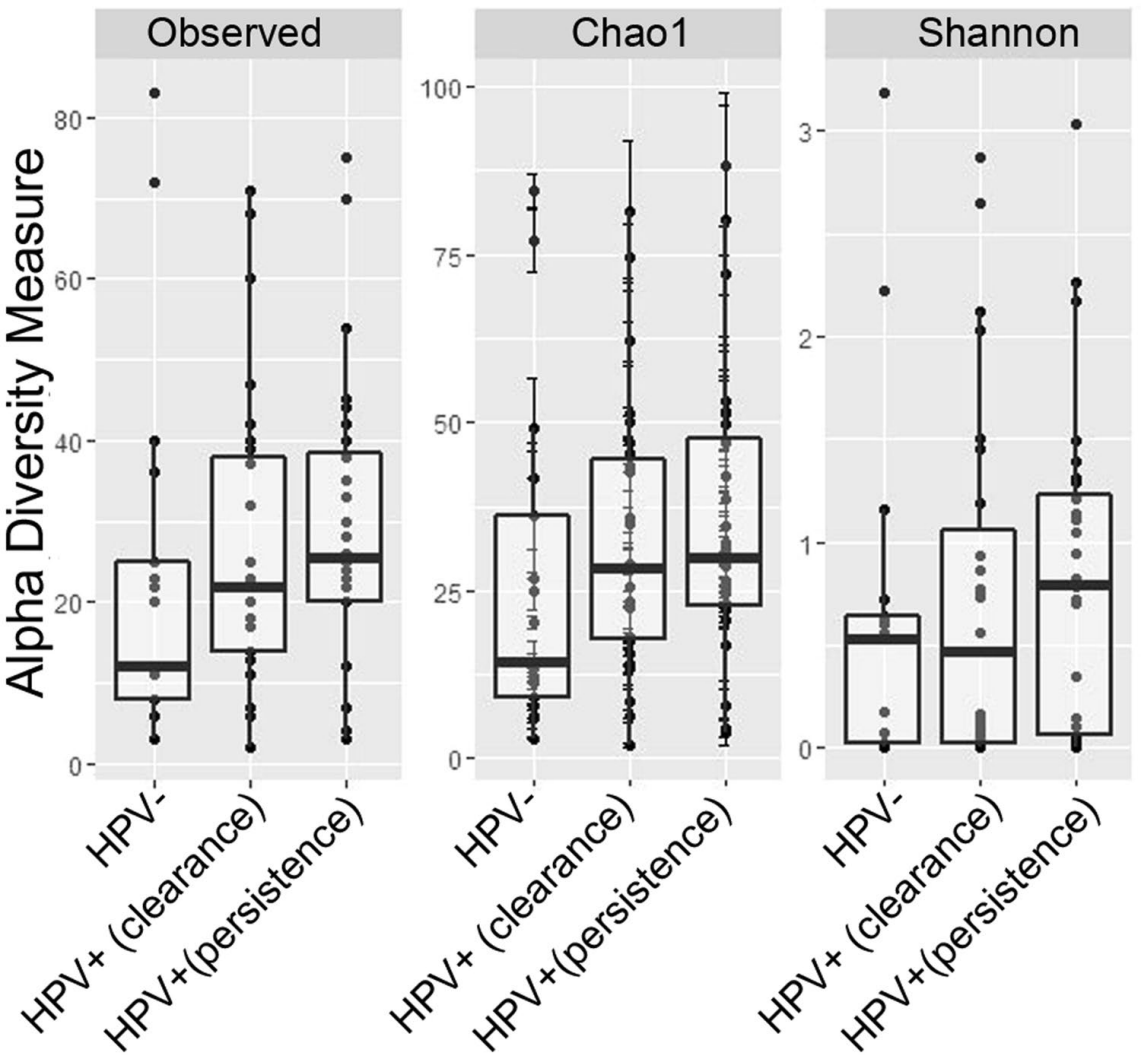
Alpha diversity measures. Box plots of observed OTUs, Chao 1, and Shannon index in the three groups of women. Pairwise comparisons by using the Wilcoxon rank sum test were not significant.

To estimate differences in microbial diversity among groups, we measured alpha diversity, based on the number of Operational Taxonomic Units (OTUs), Chao1 and Shannon indices. We observed that biodiversity was higher in the group of HPV+ women and, in particular, in samples from Persistence group compared to Control group (Fig. 1). However, the differences were not statistical significant, due to high variability observed among samples.

Evaluation of rarefaction curves (Supplementary Fig. S2A and B), calculated by number of observed OTUs for different values of the rarefaction depth for each sequenced sample showed that the curves tend to reach a plateau, indicating that the obtained sequences were sufficient to cover the real biodiversity.

**Figure 2:**
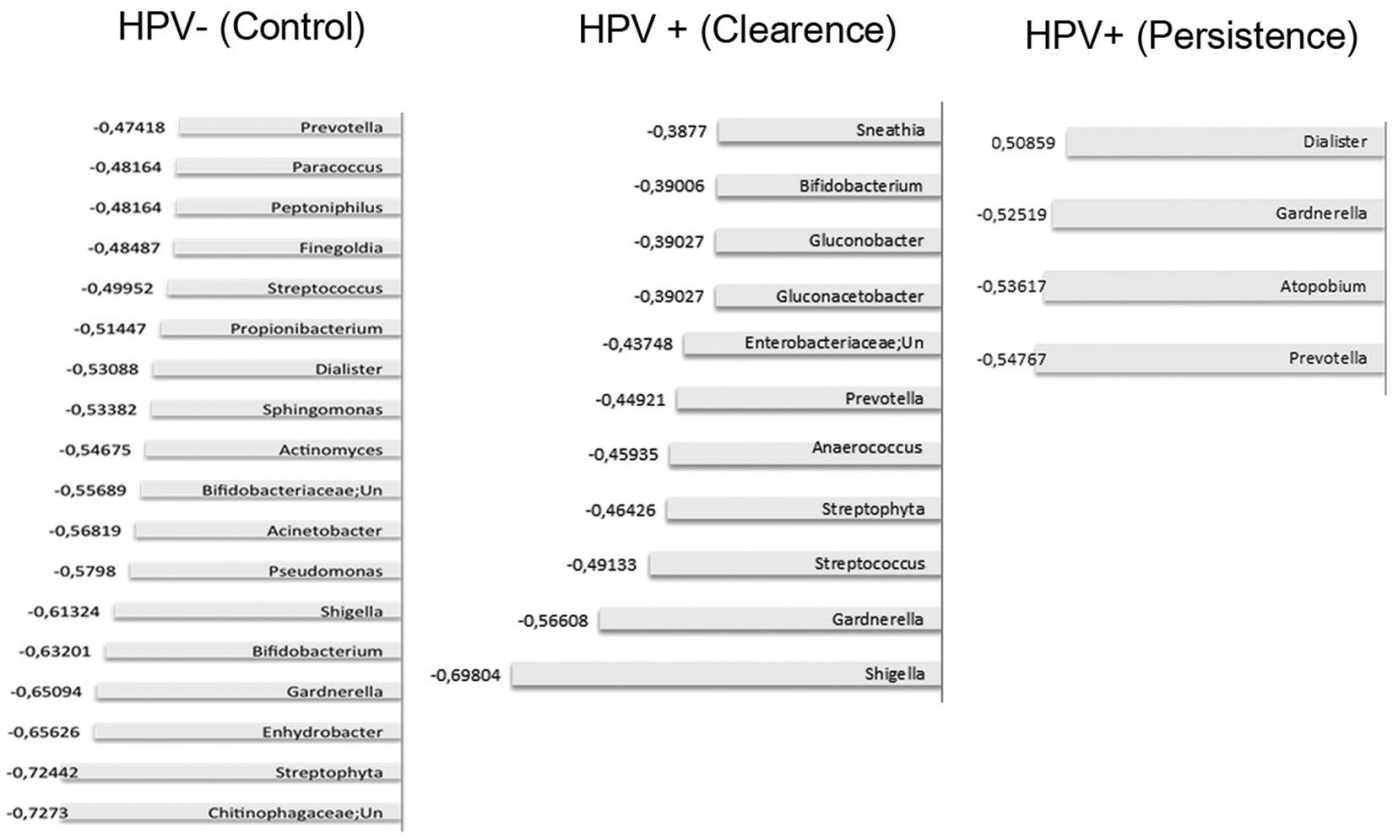
Bacterial taxa significantly correlating with reduction of *Lactobacillus*. Spearman's analysis of microbial profiles associated with *Lactobacillus* depletion in the three groups. Microbial abundance data were divided into independent data matrices (Clearance, Persistence and Controls) and pairwise Spearman’s correlation was performed with two-tailed probability of *t* for each correlation.

To investigate microbial communities populating vaginal environment following *Lactobacillus* depletion in the three groups of women, microbial abundances were divided into independent data matrices (Clearance, Persistence, Control groups) and pairwise Spearman’s correlation was performed with two-tailed probability of *t* for each correlation. This analysis indicated that few genera of anaerobic bacteria (*Prevotella*, *Gardnerella*, *Atopobium*, *Dialister*) significantly correlated with depletion in *Lactobacillus* spp. in the Persistence group. In contrast, *Streptococcus*, *Streptophita*, *Aerococcus*, and numerous genera from *Enterobacteriacee* family significantly correlated with *Lactobacillus* depletion in the Clearance and Control groups (Fig. 2).

These data strongly suggested that microbiota composition, following the depletion of protective *Lactobacillus* species, is different in the three groups and that peculiar microbial profiles may affect the outcome of viral infection.

### CST distribution in patients with different outcome of HPV infection

**Figure 3:**
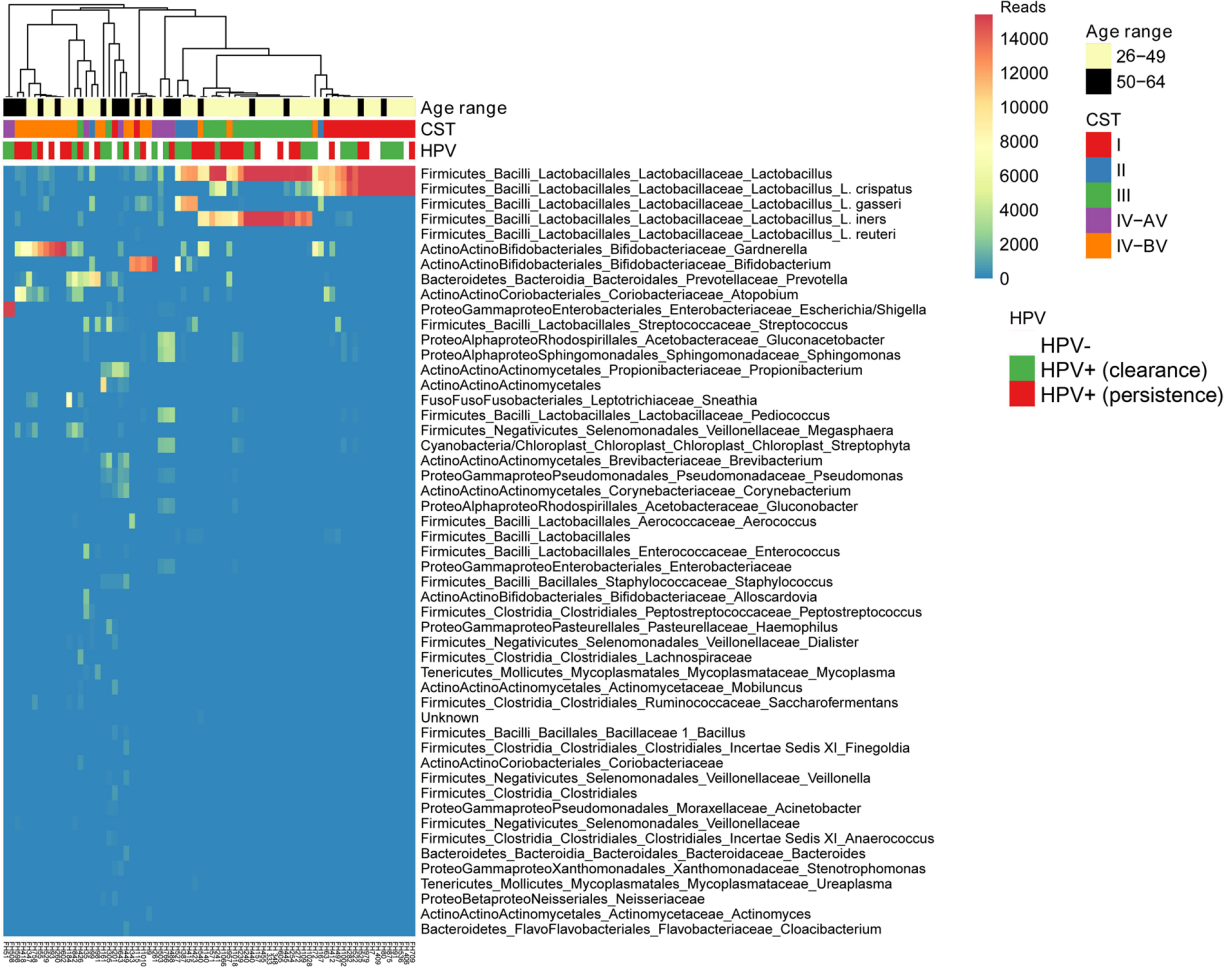
Survey of bacterial abundances in the enrolled women. Heatmap of the 50 most abundant bacterial taxa in cervico-vaginal microbiota of all enrolled women. For bacterial abundance taxa colour-scale bar indicates the number of sequence reads (from 0 up to 15400 reads). Dendogram was obtained by hierarchical clustering and was used to cluster samples of cervico-vaginal microbiota based on average distances calculated by Pearson correlation (by ClustVis tool). For each sample, we indicated age range (yellow = 26–49, and black = 50–64 age range), HPV status (white = HPV−, green = HPV+ Clearance, and red = HPV+ Persistence) of all enrolled women and CST groups (from I to IV).

Based on taxonomic distribution and accordingly with previous studies^11^, we identified four major groups of microbial communities (CSTs) which were designated as CST I, II, III, IV, according to Ravel and Mitra^7, 29, 32^. Performing hierarchical clustering analysis of the samples based on taxonomic abundances in each enrolled women we found association with HPV status, age range and CSTs (Fig. 3). CST I was dominated by *L. crispatus*, CST II by *L. gasseri*, while CST III by *L. iners*. CST IV was characterized by a paucity of *Lactobacillus* spp. (lower than 60%) and by a wide array of strict or facultative anaerobes. Most of CST IV samples were dominated by anaerobic bacteria belonging to genera *Gardnerella*, *Prevotella*, *Atopobium*, *Sneathia*, which are frequently found in women with bacterial vaginosis (BV). We named this subgroup of CST IV as CST IV-BV.

The remaining samples ascribed to CST IV showed *Lactobacillus* depletion (<60%) and a mixture of aerobic and anaerobic bacteria of the genera *Pseudomonas*, *Brevibacterium*, *Peptostreptococcus*, *Enterococcus*, *Streptococcus*, *Propionibacterium*, *Bifidobacterium*, *Shigella*. Differently than CST IV-BV, strictly anaerobic bacteria of the *Gardnerella*, *Prevotella*, *Atopobium*, *Megasphoera* genera were absent or poorly represented. This subgroup of CST IV was named CST IV-AV.

**Figure 4:**
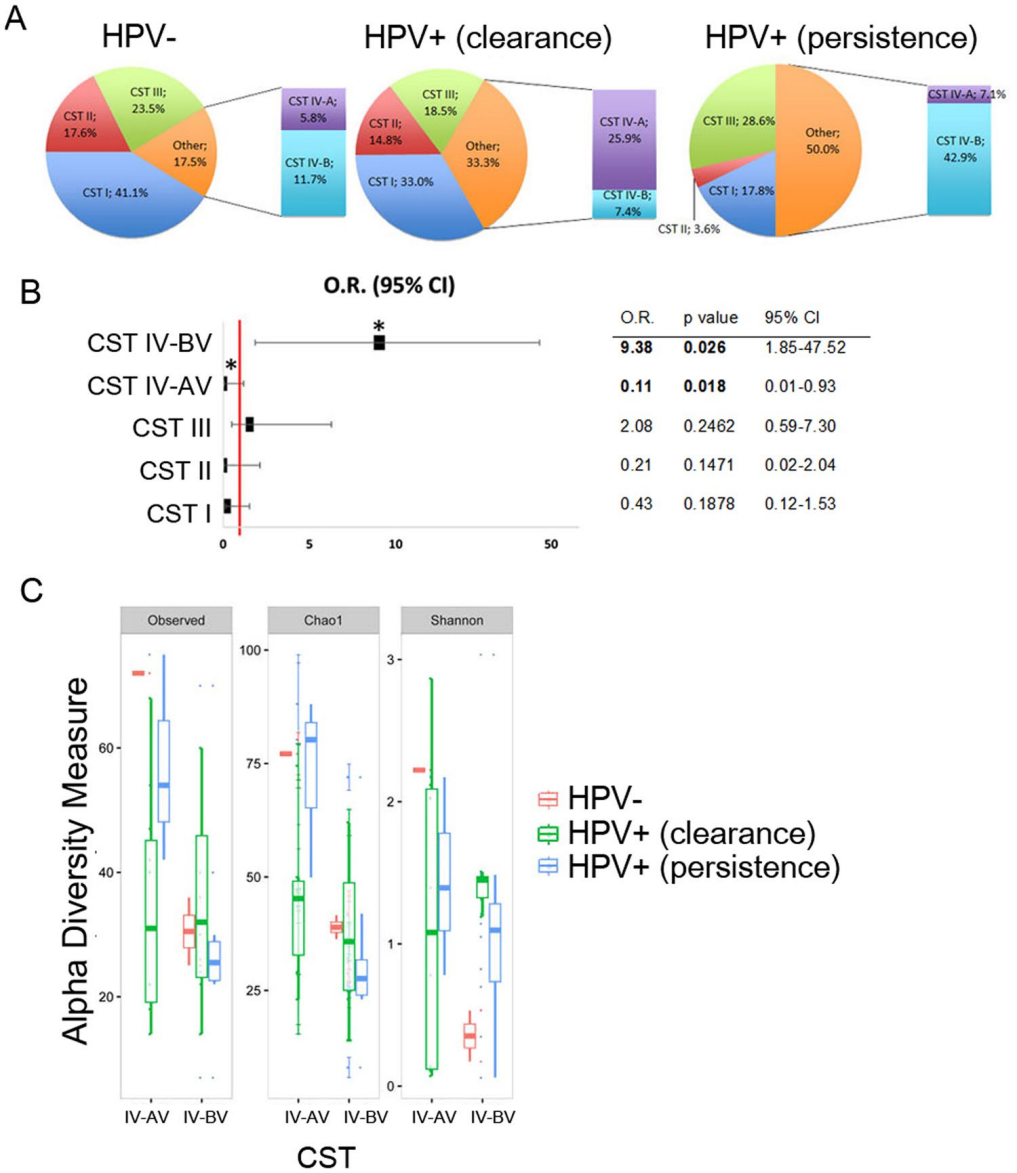
CST distribution among the groups. (**A**) Pie charts of the frequency of CSTs in the three groups. (**B**) Odds ratio. Bars indicate 95% confidence intervals. Red line indicates null value (OR = 1.0); *indicates *p* < 0.05. (**C**) Alpha diversity measure in cervico-vaginal microbiota in CST IV subgroup. Box plots of observed OTUs, Chao 1, and Shannon index in the three groups of patients. Pairwise comparisons by using the Wilcoxon rank sum test were not significant.

We observed an increased frequency of CST IV-BV (42.9%) in Persistence group compared either with Clearance group (7.4%) or with Control group (11.7%). These data (Fig. 4A and Supplementary Table S3) strongly suggested that CST IV-BV may be a risk factor for the persistence of HR-HPV infection and indeed statistical analysis revealed an odds ratio = 9.38 (95% confidence interval 1.85–47.52, *p* = 0.026; Fig. 4B). Furthermore, we observed that two women from Persistence group, annotated as CST III, also showed high percentage (≥40%) of anaerobic microbial communities typical of CST IV-BV.

To rule out that these data are mainly determined by the high frequency of CST-IV in post-menopausal women, we repeated the analysis considering only women in reproductive age exposed to HPV infection. The results obtained confirmed that CST IV-BV is significantly correlated to viral persistence in this group of women (odd ratio 7.08 at 95% confidence interval 1.31–38.33, p = 0.014).

Alpha diversity analysis of the CST-IV subtypes showed lower species richness in CST IV-BV compared to CST IV-AV (Fig. 4C), especially in Persistence group. Although the differences among the groups did not reach statistical significance, these data suggest that, in addition to the reduction in *Lactobacillus* species, only selected microbial communities (CST IV-BV) play a role in the persistence of viral infection.

**Figure 5:**
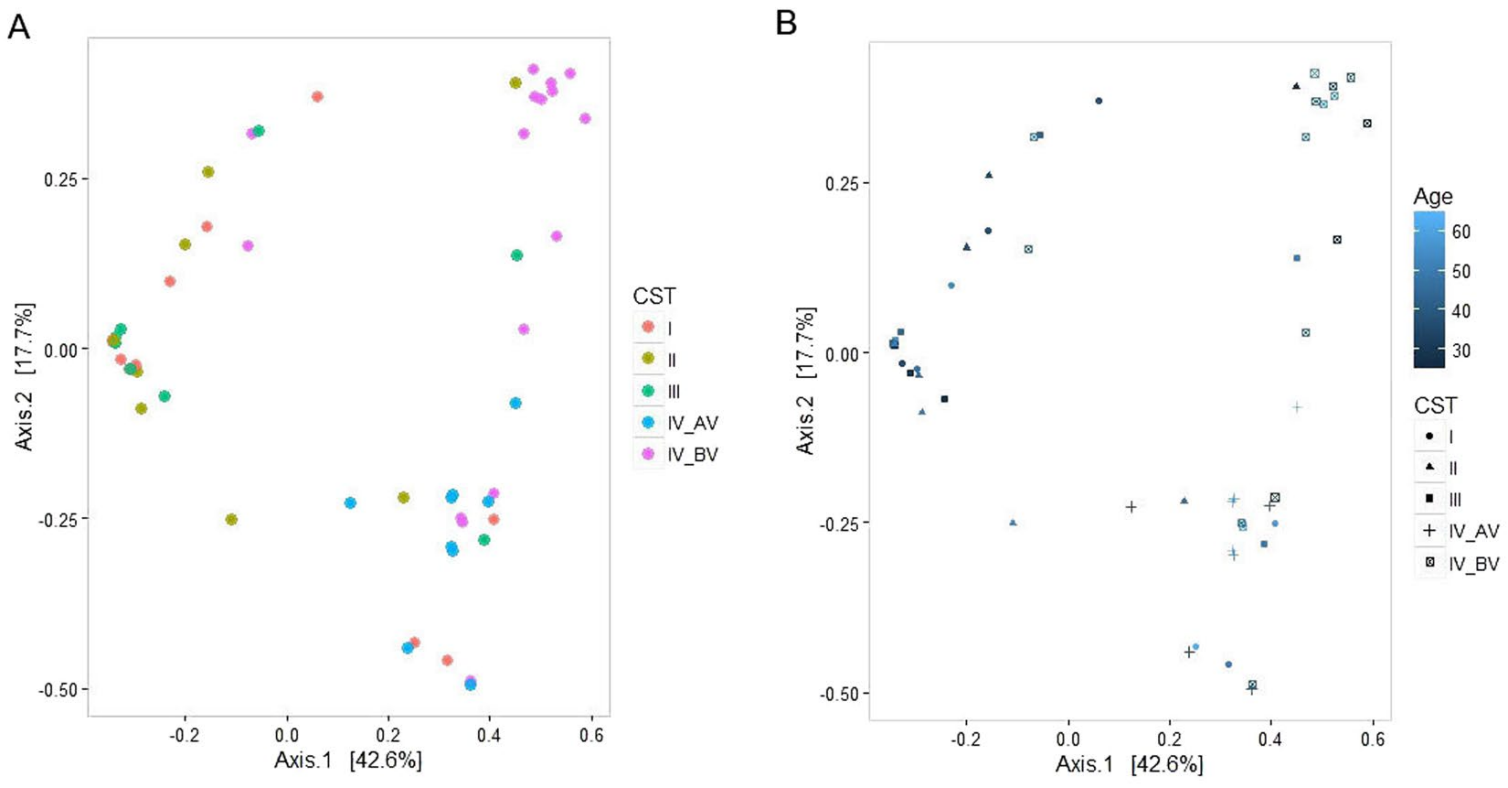
Beta diversity measure. (**A**,**B**) PCoA, based on Bray Curtis dissimilarities, correlated with CSTs and Age range. (**A**) Samples belonging to different CSTs are indicated with different colour dots. (**B**) CSTs are indicated with different forms (dots, triangles, cross-marks and squares). Age range are indicated with different blue colour-scales. p = 0.001 for CST, p = 0.002 for Age and p = 0.009 for Age:CST, respectively by PERMANOVA using the adonis() function with 999 permutations.

Finally, to evaluate variability of microbial communities among groups, we performed beta diversity analysis by using Principal Coordinates Analysis (PCoA) and Non-metric Multi Dimensional Scaling (NMDS), based on Bray-Curtis dissimilarities (Fig. 5 and Supplementary Fig. S3). By PERMANOVA analysis, we observed that samples distribution of the different groups was significantly correlated with CSTs (*p* = 0.001), age range of women (*p* = 0.009) or for both CST and age range (*p* = 0.002). In contrast, microbial diversity was not dependent on reproductive age (Supplementary Fig. S4).

### Key phylotype in persistent HPV infection

**Figure 6:**
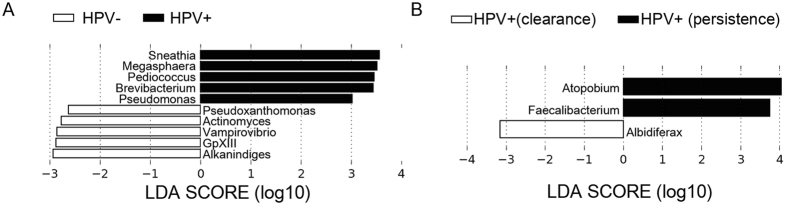
Metagenomic biomarker discovery by LEfSe analysis. Enrichment in bacterial taxa between (**A**) HPV+ and HPV− women; (**B**) HPV+ (Clearance) and HPV+ (Persistence) women. Results indicated the statistically significant taxa enrichment among groups (Alpha value = 0.05 for the factorial Kruskal-Wallis test among classes). The threshold for the logarithmic LDA score was 2.0.

To define potential metagenomic biomarkers, useful as classifiers, and to evaluate differences in abundances between assigned taxa with respect to HPV status and/or to clearance/persistence, we performed Linear Discriminant Analysis (LDA) Effect Size (LEfSe) analysis (see Methods). In agreement with previous reports we found a significant enrichment in *Sneathia* (*Leptotrichiaceae* family)^33^, *Megasphaera* (*Veillonellaceae* family) and *Pseudomonas* (*Pseudomonaceae* family)^34^ and also in *Pediococcus* (*Lactobacillaceae* family) and *Brevibacterium* (*Brevibacteriaceae* family) in the group of HR-HPV+ women compared to HPV− controls (Fig. 6A). Furthermore, considering only HPV+ women, we found enrichment in *Atopobium* as well as a lower abundance in *Faecalibacterium* genus in the Persistence group compared to the Clearance group (Fig. 6B).

### Sialidase-encoding gene from *G. vaginalis* as potential marker of viral persistence

**Figure 7:**
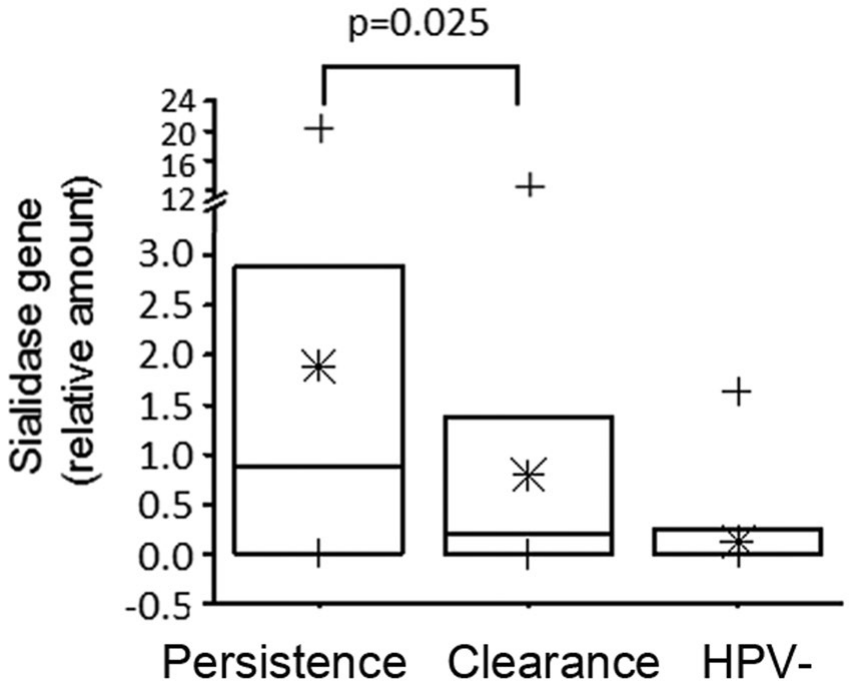
Quantitative RT-PCR for *G. vaginalis* sialidase-encoding gene. Data are expressed as relative amounts of sialidase-encoding gene. Box chart extends from SE with a lane indicating median value and an asterisk as mean value; +symbols indicate range values. Statistical analysis was performed by Mood’s Median Test and p < 0.05 was considered significant.

*Gardnerella* is one of the dominant genera in our survey. It is known that selected strains of *G. vaginalis* adopt the biofilm mode of growth as a survival strategy^35, 36^ and through symbiotic relationships with normally dormant vaginal anaerobes (*Prevotella, Atopobium*) may lead to increase of the latter. The production of sialidase is an important step in the biofilm formation^37^. To investigate whether sialidase-producing *Gardnerella* spp. were differently represented in microbial communities of the four CSTs, we studied the presence and the relative amount of sialidase-encoding gene (*GVSI*) by quantitative Real Time PCR. The amount of sialidase-encoding gene of *G. vaginalis* was significantly higher in the Persistence group compared with Clearance group (Fig. 7; Mood’s median test, MD Test p = 0.025) and especially in CST IV-BV samples (odds ratio = 12 _1.58–91.09_, 95% CI p = 0.010), indicating a strong association among species of *G. vaginalis* producing sialidase, relevant for biofilm formation, CST IV-BV and HPV persistence.

## Discussion

Metagenomics has been previously used to assess the impact of vaginal microbiota composition in HPV infection. Most of these studies showed lower proportion of *Lactobacillus* spp. and higher diversity in HPV-infected women compared to HPV-negative^32, 38^.

Brotman and co-workers^39^ performed a large longitudinal study in a cohort of 32 premenopausal women using self-sampling at twice-weekly intervals over the course of 16 weeks. The results of this study showed that CST III (dominance of *L. iners*) and IV (depletion in *Lactobacilli*) were most likely associated with HPV infection^39, 40^.

In our survey, the use of cervico-vaginal samples collected in the course of a primary screening and stored in a biological bank, gave us the opportunity to discriminate samples from women in which viral infection was cleared at one-year follow up, from those who developed persistent infection and to evaluate cervico-vaginal microbial profiles as a predictive risk factor of HPV persistence.

Microbiota characterization was performed at baseline sampling in all of HPV+ women (55/1029) involved in the study and HPV negative as control, with age between 26–64 years, in order to evaluate microbial profiles independently on the reproductive age.

In accordance with previous studies, the higher alpha diversity observed in the group of HPV+ women and especially in the Persistence group suggests that increase in species richness (compared to a *Lactobacillus*-dominated microbiota) could become a negative indicator in the more simplified vaginal communities. When compared with HPV− women, we found selected genera (*Sneathia*, *Megasphaera* and *Enterobacteriaceae)* significantly enriched in the HPV+ women. These results are in agreement with those reporting the prevalence of *Sneathia* in HPV+ patients with Squamous Intraepithelial Lesion (SIL)^34^.

The classification of cervico-vaginal microbial communities based on the CSTs reported by Ravel *et al*.^11^ allows us to define the CST IV (with *Lactobacillus* depletion) as the most represented bacterial community in HR-HPV infected women compared to negative controls. However, two subgroups of CST IV could be identified in our records. Few strictly anaerobic bacteria of the *Gardnerella*, *Prevotella*, *Atopobium*, *Megasphera*, *Dialister* genera dominated CST IV in women developing persistent infection. We referred this type of CST as CST IV-BV, since the same bacterial communities are frequently found in women with bacterial vaginosis (BV). A mixture of aerobic and anaerobic bacteria including *Pseudomonas*, *Brevibacterium*, *Peptostreptococcus*, *Enterococcus*, *Streptococcus*, *Propionibacterium*, *Bifidobacterium* and *Shigella*, namely CST IV-AV subtype, was prevalent in the Clearance group. The differences among the groups in bacterial communities populating vaginal environment, following depletion in *Lactobacillus* species, were evident through the Spearman's correlation analysis. Beta diversity analysis showed that, microbial variability among samples is correlated with CSTs and age range of women. However, the adoption of the CST classification proposed by Ravel^11^ allowed us to perform a risk association analysis, and despite the limited sample size, we found a significant association between CST IV-BV and the persistence of HR-HPV infection, which was not dependent on the reproductive age. Unfortunately, the lack of additional clinical and behavioural information of the enrolled women did not allow the revealing of further correlations considering CST subgroups and in particular with CST IV-BV.

However, we observed a very low alpha diversity in the CST IV-BV in women of the Persistence group, indicating that only a limited number of bacterial genera are likely involved in viral persistence. Based on our results, we suggest that CST IV-BV subtype play a role in viral persistence. To define potential metagenomic biomarkers of persistent infection, we identified *Atopobium* as key phylotype in the Persistence group compared to the Clearance group.

Finally, to define molecular markers associated with viral persistence, we evaluated the relative amount of sialidase-encoding gene from *Gardnerella vaginalis* species in the three groups of women. As known, selected strains of *G. vaginalis* are able to form biofilm and the production of sialidase is an important step in the biofilm formation^37^. This enzyme facilitates the destruction of the protective mucus layer on the vaginal epithelium allowing resistant adhesion of bacteria, which can start to form biofilm^35, 36^. The observed higher amount of sialidase-encoding gene in the Persistence group compared to other groups and in particular the prevalence in CST IV-BV suggest that sialidase-producing *G. vaginalis* strains are likely involved in formation of biofilm entrapping anaerobic pathobionts, such as *Prevotella* and *Atopobium*, favouring their overgrowth^41^, and that these biofilms may contribute to viral persistence.

*Atopobium vaginae*, which is significantly more represented in microbiota of women in Persistence group compared to Clearance group, may play a role in the disruption of epithelial barriers^42^, favouring either HPV infections or viral dissemination. A recent study reports that anaerobes as *Prevotella, Gardnerella, Atopobium, Sneathia*, induce a strong inflammation in vaginal environment with high recruitment of T-helper 17 lymphocytes, which favours HIV infection^26^. Although no differences in lymphoid population were associated with clearance or persistence of HPV infection^43^, a high concentration of inflammation markers (IP-10 and MIG chemokines) was found in the vaginal environment of women who cleared the virus. The differential inflammatory potential of bacterial taxa populating vaginal environment in the Clearance and Persistence groups need to be elucidated. While a high inflammatory environment could facilitate viral clearance, immune-modulating bacterial genera, such as *Faecalibacterium* ^44, 45^, could play a role in the progression of disease in later stages of infection. Further investigations are needed to establish the role of bacterial biofilms and/or the induction of immunomodulation in HPV disease.

Finally, although we could not ascertain microbiota stability in our patients during one-year intercourse, we can conclude that the early cervico-vaginal microbiota characterization may help to discriminate women at risk to develop persistent HPV infection and provide useful insights to develop new therapeutic strategies, modifying the vaginal microbiota ecosystem.

## Methods

### Population study

A total of 1029 women, resident in Florence district (Italy) in both reproductive and post-menopausal age, who never received HPV vaccine, were enrolled in a HPV DNA based cervical cancer screening program in the contest of pilot study aimed to evaluate efficacy of HPV testing for the detection of invasive cervical cancer and cervical intrahepithelial neoplasia. The study was approved by the Ethics Committee of Azienda USL 10, Florence, Italy (Protocol ref. 263/2009). The protocol and the methods were performed in accordance with relevant guidelines and regulations. Informed consent for using biological samples for scientific investigations, including microbiota characterization, was obtained from all women at the time of the first screening.

All enrolled women were of Caucasian ethnicity. Group Age ranged between 26–64 years. Exclusion criteria were: (i) pregnancy (ii) HIV infection (ii) immunodeficiency-related diseases, including AIDS. Abstinence from sex and from using vaginal antibiotics and/or vaginal washes was required until three days before collection.

Cervico-vaginal samples were collected in Specimen Transport Medium (STM; Qiagen, Milano Italy) by trained midwives at Istituto per lo Studio e la Prevenzione Oncologica (ISPO; Florence, Italy).

HR-HPV status was determined through HR-HC2 assay (see paragraph below) and the remaining samples were stored at −80 °C for bacterial genomic DNA extraction.

Samples from all of HPV+ women (55/1029) and from 17 selected age-matched HPV− women (who gave consensus for scientific investigations) were used for microbiota characterization. All information obtained by consensus are summarized in Table 1. No further information could be acquired later.

HR-HPV+ women were not directly referred to colposcopy but were triaged according to cytological testing. If the result of this test was abnormal (Atypical Squamous Cells of Undetermined Significance -ASC-US- or a more severe lesion) the women were immediately referred to colposcopy. If cytology was normal, the women were invited to repeat a new HPV test after one year, following the HTA protocol^46^. When Atypical Squamous Cells of Undetermined Significance (ASC-US) or a more severe lesion was ascertained, women were referred for colposcopy to define the grade (low or high) of lesions, according to the Italian cervical cancer screening protocol (Ministero della Salute Italiana, Italy). Furthermore, HPV genotyping was used to confirm the viral persistence.

Supplementary Table S1 shows the distribution of age, HPV status at baseline screening and at follow-up, the genotype(s) of HPV for each woman, as well as the colposcopy report.

### Bacterial DNA extraction

Bacterial genomic DNA was extracted from the cervico-vaginal samples stored at −80 °C, by using a QiAmp Mini DNA kit (Qiagen, Milano, Italy) as previously described^38^. Quality control was carried out by gel electrophoresis and measuring ng/µl of DNA and 260/280 OD at Nanodrop 1000 (Thermo Scientific).

### HPV-assay and HPV genotyping

HPV DNA test was performed according to manufacturer’s guidelines using the Hybrid Capture 2 assay (HC2; Qiagen, Milano, Italy).

The HC2 assay is a nucleic acid hybridization assay with signal amplification using microplate chemiluminescence for the qualitative detection of thirteen high-risk types (16, 18, 31, 33, 35, 39, 45, 51, 52, 56, 58, 59, 68) of HPV DNA in cervical specimens.

Genotyping was performed on HR-HC2 positive samples. Specific genotyping, which identifies thirty-one HPV types, was performed by amplifying the target DNA with PGMY09/11 and HLA biotinylated primers, using the validated method of the WHO HPV LabNet group^47^.

### Pyrosequencing and data analysis

We amplified the 16S rRNA gene by using primer set specific for V3-V5 hypervariable regions, and the FastStart High Fidelity PCR system (Roche Life Science, Milano, Italy) as described in Strati *et al*.^48^. The 454 pyrosequencing was carried out on the GS FLX+ system using the XL+ chemistry following the manufacturer’s recommendations.

Pyrosequencing resulted in a total of 2,112,426 16S rDNA reads with a mean of 26,405 sequences per sample. Average sequence lengths were 589 nt (±SD 28.4), 591 nt (±SD 25.7) and 594 nt (±SD 24.39) for the first, second and third run, respectively. Raw 454 files were demultiplexed using Roche’s.sff file software, and made available at the European Nucleotide Archive (http://www.ebi.ac.uk/ena/data/view/PRJEB18720) under the accession study PRJEB18720. Pre-processing of the reads was performed using the MICCA pipeline (version 0.1, http://www.micca.org). Trimming of the primers and quality filtering were performed using micca-preproc, truncating reads shorter than 280 nt (quality threshold = 18; http://micca.org/docs/0.1/command_ref/micca_preproc.html; Supplementary Table S4). *Denovo* sequence clustering, chimera filtering and taxonomy assignment were performed by micca-otu-denovo (parameters −s 0.97 −c). Operational Taxonomic Units (OTUs) were assigned by clustering the sequences with a threshold of 97% pair-wise identity. The representative sequences were classified using the RDP classifier version 2.7 against RDP 11 database (update 5) of 16S rRNA. Template-guided multiple sequence alignment was performed using PyNAST (version 0.1) against the multiple alignment of the Greengenes 16S rRNA gene database (release 13_05) andfiltering at 97% similarity. A phylogenetic tree was inferred using FastTree and micca-phylogeny (parameters: -a template-template-min-perc 50). Rarefaction was performed in order to reduce the sampling heterogeneity. A total of 15,400 sequences per sample was obtained.

Observed OTUs, Chao1 index, Shannon entropy (indicators of alpha diversity) and Bray-Curtis dissimilarities (indicators of beta diversity) were calculated using the phyloseq package of the R software suite.

Community State Type (CST) of each cervico-vaginal microbiota was defined considering the relative abundance of *Lactobacillus* spp. as >60%, and aerobic and anaerobic bacteria ranging from 14 to 40%.

To evaluate the differences in overall bacterial community composition, Principal Coordinates Analysis (PCoA) and Non-metric Multidimensional Scaling (NMDS), based on Bray-Curtis dissimilarities were performed. The significance of between-groups differentiation (CSTs, Age range, reproductive and menopausal age) was assessed by PERMANOVA, Adonis() function, using the R package vegan with 999 permutations.

By Clustvis tool^49^, heatmap of the relative abundances of bacterial taxa was generated. Variables such as HPV status, CST, age range of the women were associated with the respective microbiota sample. Heatmap was supported by a dendrogram obtained with hierarchical clustering of the samples and based on average distances among samples calculated by Pearson correlation.

### Quantitative Real Time PCR of sialidase-encoding gene from *Gardnerella vaginalis*

Quantitative Real Time PCR (RT q-PCR) of sialidase-encoding gene (*GVSI)* was performed by using Platinum TaqUniversal SYBR® green supermix (InvitrogenBiorad) and primers specific for *G. vaginalis* sialidase-encoding gene (Fw-5′GGGTTTATGCACACGCTT TT-3′ and Rv-5′GAAAATGCAGACAACGC AGA-3′)^50^ and universal bacterial 16 S rRNA gene (from *E. coli*; Fw-5′AGAGTTTGATCCTGGCTCAG-3′ and Rv-5′GGACTACCAGGGTATCTAAT-3′) as reference of the total bacterial community^51^. RT q-PCR was performed in Applied Biosystem 7900 instrument following these conditions: 50 °C for 2′; 95 °C for 10′, 40 cycles of denaturation at 95 °C for 15″, annealing at 55 °C for 30″, and extension at 68 °C for 30″ followed by a dissociation stage :95° for 15″; 60° for 15″ and 95° for 15″ with a rampe rate of 2%. Samples with a melting temperature (T_m_) value at 84° for sialidase-encoding gene (n = 26) and between 87 and 91 °C for the 16S r RNA gene were considered positive (n = 55).

Results are reported as relative amount of DNA calculated as 2^−ΔCT^, with ΔCT = CT _sialidase_ − CT _16S_.

### Statistical analysis

Metagenomic biomarker discovery and related statistical significance were assessed using relative taxonomic abundances analysed according to the Linear Discriminant Analysis (LDA) Effect Size (LEfSe) method^52^. In LEfSe, Kruskal–Wallis rank-sum test is used to identify features with significantly different taxa abundances among groups, and LDA to calculate the size effect of each feature. An alpha significance level of 0.05, either for the factorial Kruskal-Wallis test among classes or for the pairwise Wilcoxon test between subclasses, and a size-effect threshold of 2.0 on the logarithmic LDA score were used for discriminative microbial biomarkers.

For correlation analysis, microbiota abundance data were divided into independent data matrices (Clearance group, Persistence group and HPV− group, as control). The correlation coefficients and significant negative correlations (p < 0.05) between *Lactobacillus* abundance data and all the other taxa were calculated using the pairwise Spearman’s correlation and two-tailed probability of t for each correlation.

Odd ratio with 95% confidence interval was calculated to correlate CSTs with HPV clearance and persistence in all women population and considering also the only women in reproductive age. P < 0.05 was considered statistically significant.

Statistical analysis of RTq-PCR of sialidase-encoding gene was performed with non parametric Mood’s Median test (MDTest) and Wilcoxon Signed Rank test using the Origin software. P < 0.05 was considered statistically significant.

## Electronic supplementary material

Supplementary Information

Supplementary Dataset 2

Supplementary Dataset 4
