# Supplementary material for: Characterization of cervico-vaginal microbiota in women developing persistent high-risk Human Papillomavirus infection
Source: Sci Rep. 2017 Aug 31;7:10200. doi: 10.1038/s41598-017-09842-6 (PMC5579045; doi:10.1038/s41598-017-09842-6)
Supplement: Supplementary file 1 — Supplementary Information [file 41598_2017_9842_MOESM1_ESM.pdf]

Supplementary Information

Characterization of cervico-vaginal microbiota in women developing persistent high-risk Human Papillomavirus infection

Monica Di Paola[1], Cristina Sani[2], Ann Maria Clemente[3], Anna Iossa[4], Eloisa Perissi[3], Giuseppe Castronovo[3], Michele Tanturli[6], Damariz Rivero[5], Federico Cozzolino[6], Duccio Cavalieri[5], Francesca Carozzi[2*], Carlotta De Filippo[7*], Maria Gabriella Torcia[3*]

1. Dipartimento di Neuroscienze, Psicologia, Area del Farmaco e Salute del Bambino (NEUROFARBA), Università di Firenze, AOU Meyer;
2. S.C. Screening e Prevenzione Secondaria, Istituto per lo Studio e la Prevenzione Oncologica (ISPO) Firenze;
3. Dipartimento di Medicina Sperimentale e Clinica, Università di Firenze;
4. Laboratorio Regionale HPV e Biologia Molecolare, LRPO, Istituto per lo Studio e la Prevenzione Oncologica (ISPO) Firenze;
5. Dipartimento di Biologia, Università di Firenze;
6. Dipartimento di Scienze Biomediche Sperimentali e Cliniche, Università di Firenze;
7. Istituto di Biologia e Biotecnologia Agraria (IBBA), Consiglio Nazionale delle Ricerche CNR), Pisa

[*] Corresponding authors

Correspondence to:

Maria Gabriella Torcia, MD, Dipartimento di Medicina Sperimentale e Clinica, University of Florence, Florence Italy

e-mail: maria.torcia@unifi.it

Carlotta De Filippo, PhD, Istituto di Biologia e Biotecnologia Agraria (IBBA), National Research Council (CNR), via Moruzzi, 1, Pisa, Italy.

e-mail: carlotta.defilippo@ibba.cnr.it

Francesca Carozzi, PhD, Laboratorio Regionale HPV e Biologia Molecolare, ISPO, Florence, Italy

e-mail: f.carozzi@ispo.toscana.it

Supplementary Table

Supplementary Table S1. Characteristics of the enrolled women. Age, HPV status, either at baseline screening or at one-year follow-up, and CSTs of cervico-vaginal microbiota from each enrolled women are reported.

ID woman	Age	HPV status at baseline screening	HPV status at 1-year follow-up	HPV types at baseline screening	Colposcopy at follow-up	CST
FH 333	41	HPV negative	HPV negative	-		III
FH 348	43	HPV negative	HPV negative	-		III
FH 406	42	HPV negative	HPV negative	-		I
FH 409	39	HPV negative	HPV negative	-		I
FH167	35	HPV negative	HPV negative	-		II
FH201	57	HPV negative	HPV negative	-		I
FH260	56	HPV negative	HPV negative	-		IV_BV
FH343	43	HPV negative	HPV negative	-		IV_BV
FH445	55	HPV negative	HPV negative	-		III
FH455	27	HPV negative	HPV negative	-		III
FH497	48	HPV negative	HPV negative	-		I
FH503	35	HPV negative	HPV negative	-		IV_AV
FH529	28	HPV negative	HPV negative	-		II
FH63	54	HPV negative	HPV negative	-		I
FH7	41	HPV negative	HPV negative	-		I
FH9	50	HPV negative	HPV negative	-		I
FH99	44	HPV negative	HPV negative	-		II
FH1002	36	HPV positive	cleared HPV infection	16		I
FH1028	44	HPV positive	cleared HPV infection	35		III
FH109	42	HPV positive	cleared HPV infection	31		III
FH115	63	HPV positive	cleared HPV infection	70		I
FH161	59	HPV positive	cleared HPV infection	58		IV_AV
FH240	29	HPV positive	cleared HPV infection	18, 51		III
FH241	45	HPV positive	cleared HPV infection	16		III
FH261	40	HPV positive	cleared HPV infection	52		IV_AV
FH282	41	HPV positive	cleared HPV infection	61, 62		I
FH305	49	HPV positive	cleared HPV infection	16		III
FH35	41	HPV positive	cleared HPV infection	68		IV_AV
FH387	37	HPV positive	cleared HPV infection	16		II
FH415	49	HPV positive	cleared HPV infection	No typeable		II
FH440	50	HPV positive	cleared HPV infection	45		II
FH508	61	HPV positive	cleared HPV infection	No typeable		IV_AV
FH51	58	HPV positive	cleared HPV infection	No typeable		IV_AV
FH522	36	HPV positive	cleared HPV infection	52		I

FH527	52	HPV positive	cleared HPV infection	52		II
FH536	36	HPV positive	cleared HPV infection	18, 59		I
FH643	64	HPV positive	cleared HPV infection	39		IV_AV
FH72	38	HPV positive	cleared HPV infection	67		I
FH758	43	HPV positive	cleared HPV infection	16		IV_BV
FH766	64	HPV positive	cleared HPV infection	66		IV_AV
FH80	54	HPV positive	cleared HPV infection	56		I
FH842	26	HPV positive	cleared HPV infection	18, 53		IV_BV
FH875	30	HPV positive	cleared HPV infection	No typeable		I
FH91	47	HPV positive	cleared HPV infection	31		I
FH1010	33	HPV positive	persistent HPV infection	56	CIN1; condiloma	IV-BV
FH1018	28	HPV positive	persistent HPV infection	58, 59	CIN1; condiloma	III
FH1066	37	HPV positive	persistent HPV infection	52		III
FH140	43	HPV positive	persistent HPV infection	31, 42, 54		III
FH157	29	HPV positive	persistent HPV infection	51		III
FH184	45	HPV positive	persistent HPV infection	53		IV_BV
FH239	29	HPV positive	persistent HPV infection	51		III
FH254	30	HPV positive	persistent HPV infection	31	CIN1; Condiloma	III
FH272	31	HPV positive	persistent HPV infection	31, 59		III
FH347	26	HPV positive	persistent HPV infection	59		IV_BV
FH412	37	HPV positive	persistent HPV infection	56, 68, 84		I
FH418	56	HPV positive	persistent HPV infection	52		IV_BV
FH423	46	HPV positive	persistent HPV infection	52	CIN1; condiloma	II
FH426	51	HPV positive	persistent HPV infection	39	CIN1; condiloma	III
FH449	49	HPV positive	persistent HPV infection	No typeable	CIN2	IV_BV
FH488	54	HPV positive	persistent HPV infection	16, 58, 70		IV_AV
FH525	52	HPV positive	persistent HPV infection	52		I
FH540	51	HPV positive	persistent HPV infection	40, 70		IV_BV
FH55	53	HPV positive	persistent HPV infection	18		IV_BV
FH571	31	HPV positive	persistent HPV infection	16		III
FH598	51	HPV positive	persistent HPV infection	68		IV_BV
FH602	39	HPV positive	persistent HPV infection	52		IV_BV
FH605	44	HPV positive	persistent HPV infection	54, 56	CIN2	I
FH709	36	HPV positive	persistent HPV infection	31, 51, 54, 59		I
FH93	45	HPV positive	persistent HPV infection	54		IV_BV
FH957	45	HPV positive	persistent HPV infection	59		IV_BV
FH979	47	HPV positive	persistent HPV infection	45, 58		I
FH981	32	HPV positive	persistent HPV infection	31	CIN1; condiloma	IV_BV

Supplementary Table 3. CST distribution among the groups.

	**HPV- Control group N=17**	**%**	**HPV+ Clearance group N=27**	**%**	**HPV+ Persistence group N=28**	**%**
CST I	7	41.1	9	33	5	17.8
CST II	3	17.6	4	14.8	1	3.6
CST III	4	23.5	5	18.5	9	32.1
CST IV-AV	1	5.8	7	25.9	1	3.5
CST IV-BV	2	11.7	2	7.4	12	42.9

Data files

Supplementary Table 2. Cervico-vaginal microbiota profiles at different taxonomic levels. Number of reads at (A) phylum, (B) family and (C) genus level obtained for each sequenced sample, (D) all taxonomy, (E) Number of reads for *Lactobacillus* species was reported.

Supplementary Table 4. Reports of (A) 454 platform sequencing, (B) mica pipeline preprocessing of the reads, and (C) number of sequenced reads pre- and post filtering.

Supplementary Figure

A)

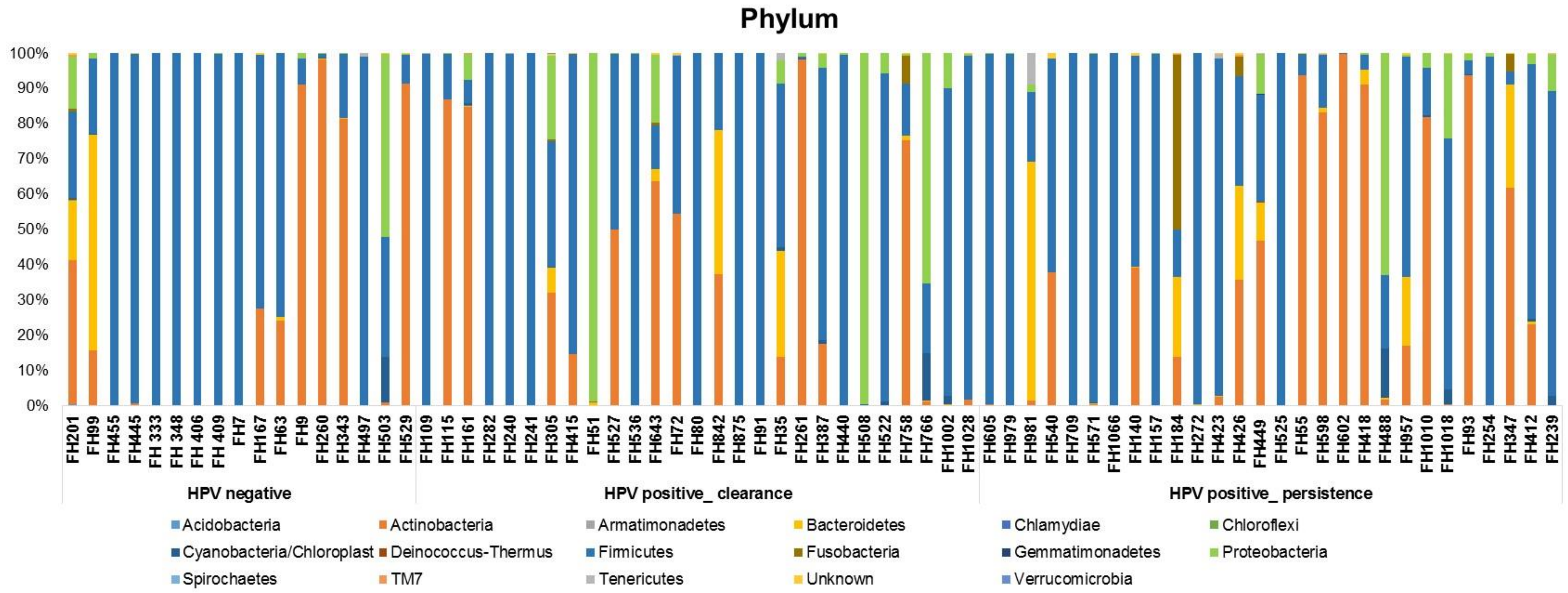

B)

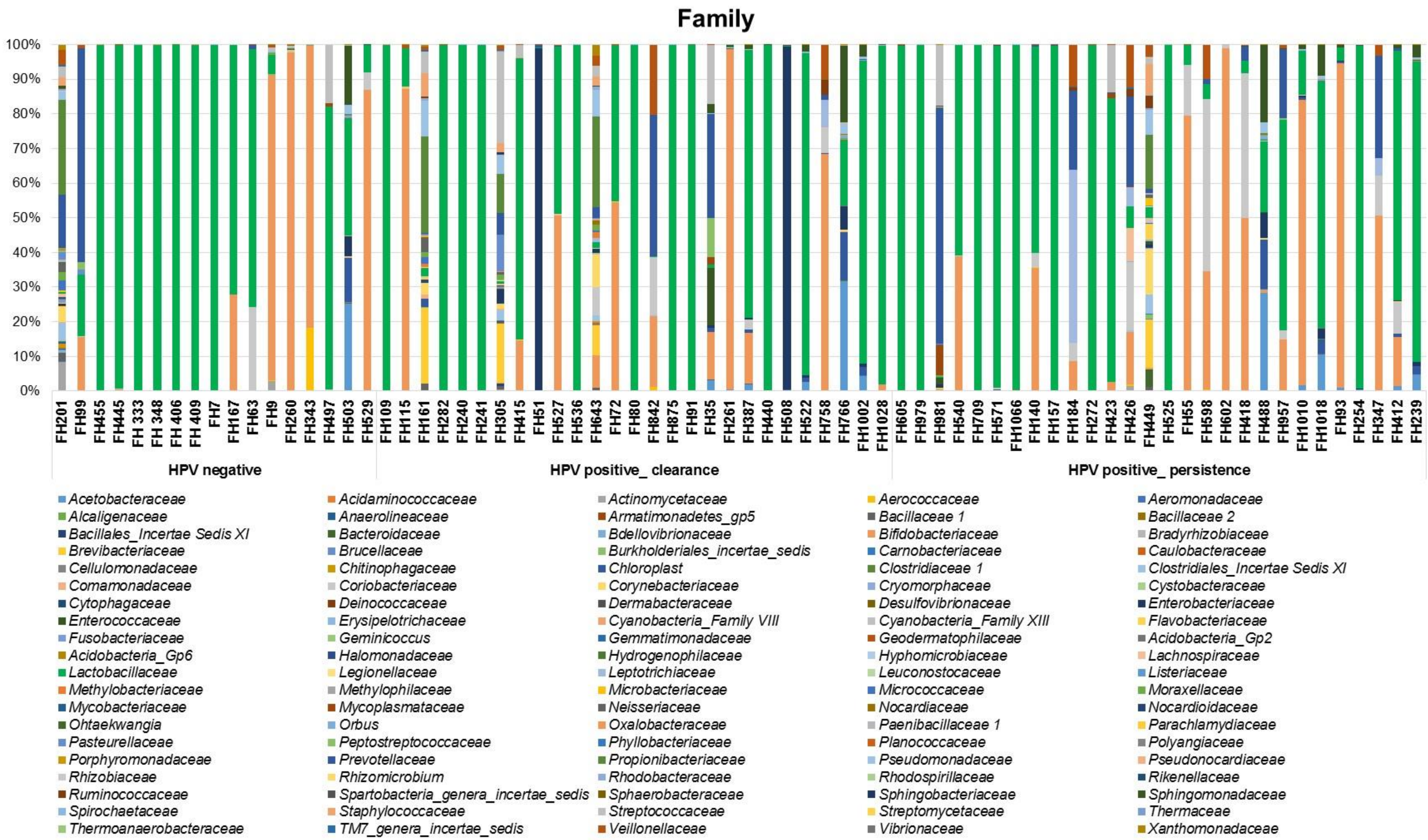
Family
100%
90%
80%
70%
60%
50%
40%
30%
20%
10%
0%
FH201 FH99 FH455 FH445 FH 333 FH 348 FH 406 FH 409 FH7 FH167 FH63 FH9 FH260 FH343 FH497 FH503 FH529
FH109 FH115 FH161 FH282 FH240 FH241 FH305 FH415 FH51 FH527 FH536 FH643 FH72 FH80 FH842 FH875 FH91 FH35 FH261 FH387 FH440 FH508 FH522 FH758 FH766 FH1002 FH1028
FH605 FH979 FH981 FH540 FH709 FH571 FH1066 FH140 FH157 FH184 FH272 FH423 FH426 FH449 FH525 FH55 FH598 FH602 FH418 FH488 FH957 FH1010 FH1018 FH93 FH254 FH347 FH412 FH239
HPV negative
HPV positive_ clearance
HPV positive_ persistence
Acetobacteraceae
Acidaminococcaceae
Actinomycetaceae
Aerococcaceae
Aeromonadaceae
Alcaligenaceae
Anaerolineaceae
Armatimonadetes_gp5
Bacillaceae 1
Bacillaceae 2
Bacillales_Incertae Sedis XI
Bacteroidaceae
Bdellovibrionaceae
Bifidobacteriaceae
Bradyrhizobiaceae
Brevibacteriaceae
Brucellaceae
Burkholderiales_incertae_sedis
Carnobacteriaceae
Caulobacteraceae
Cellulomonadaceae
Chitinophagaceae
Chloroplast
Clostridiaceae 1
Clostridiales_Incertae Sedis XI
Comamonadaceae
Coriobacteriaceae
Corynebacteriaceae
Cryomorphaceae
Cystobacteraceae
Cytophagaceae
Deinococcaceae
Dermabacteraceae
Desulfovibrionaceae
Enterobacteriaceae
Enterococcaceae
Erysipelotrichaceae
Cyanobacteria_Family VIII
Cyanobacteria_Family XIII
Flavobacteriaceae
Fusobacteriaceae
Geminicoccus
Gemmatimonadaceae
Geodermatophilaceae
Acidobacteria_Gp2
Acidobacteria_Gp6
Halomonadaceae
Hydrogenophilaceae
Hyphomicrobiaceae
Lachnospiraceae
Lactobacillaceae
Legionellaceae
Leptotrichiaceae
Leuconostocaceae
Listeriaceae
Methylobacteriaceae
Methylophilaceae
Microbacteriaceae
Micrococcaceae
Moraxellaceae
Mycobacteriaceae
Mycoplasmataceae
Neisseriaceae
Nocardiaceae
Nocardioidaceae
Ohtaekwangia
Orbus
Oxalobacteraceae
Paenibacillaceae 1
Parachlamydiaceae
Pasteurellaceae
Peptostreptococcaceae
Phyllobacteriaceae
Planococcaceae
Polyangiaceae
Porphyromonadaceae
Prevotellaceae
Propionibacteriaceae
Pseudomonadaceae
Pseudonocardiaceae
Rhizobiaceae
Rhizomicrobium
Rhodobacteraceae
Rhodospirillaceae
Rikenellaceae
Ruminococcaceae
Spartobacteria_genera_incertae_sedis
Sphaerobacteraceae
Sphingobacteriaceae
Sphingomonadaceae
Spirochaetaceae
Staphylococcaceae
Streptococcaceae
Streptomycetaceae
Thermaceae
Thermoanaerobacteraceae
TM7_genera_incertae_sedis
Veillonellaceae
Vibrionaceae
Xanthomonadaceae

C)

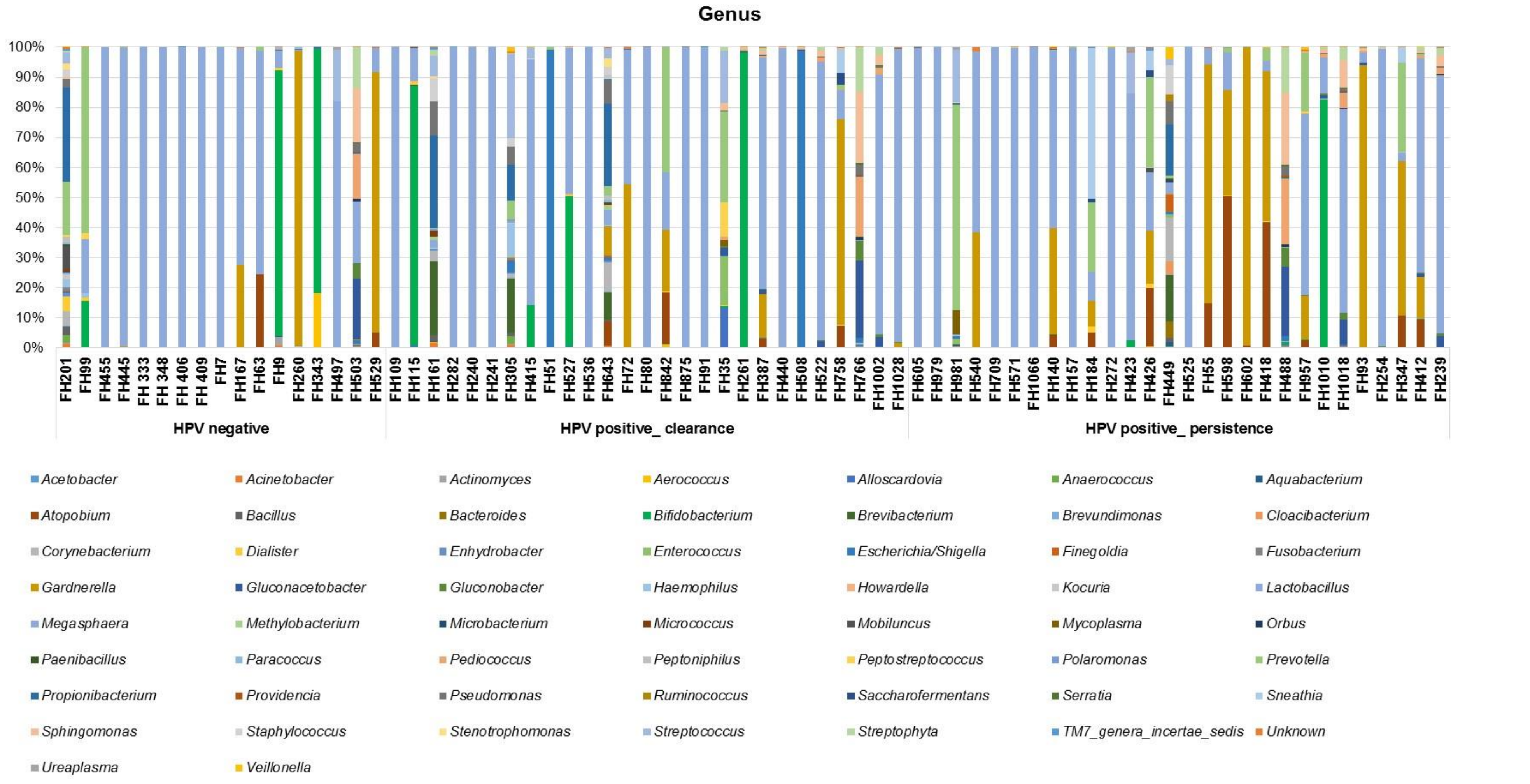

Supplementary Fig. S1. Relative abundances of the cervico-vaginal microbiota at different taxonomic levels. Barplots of (A) phylum, (B) family and (C) genus relative abundance of HPV-, HPV+_Clearance and HPV+_Persistence groups.

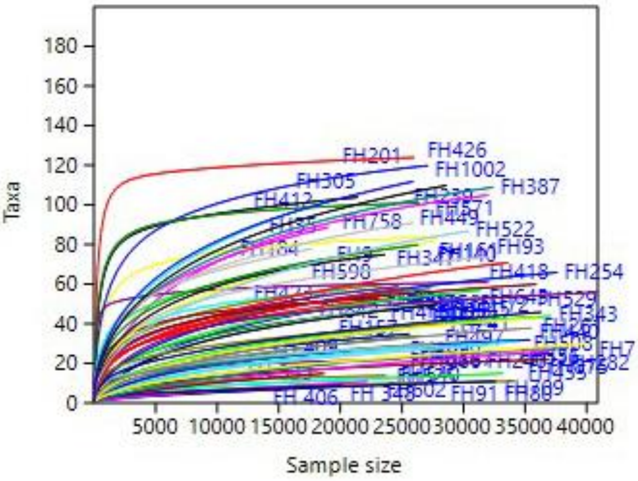

Supplementary Fig. S2. Rarefaction curves based on number of observed OTUs for each sequenced sample, obtained by PAST3 tool.

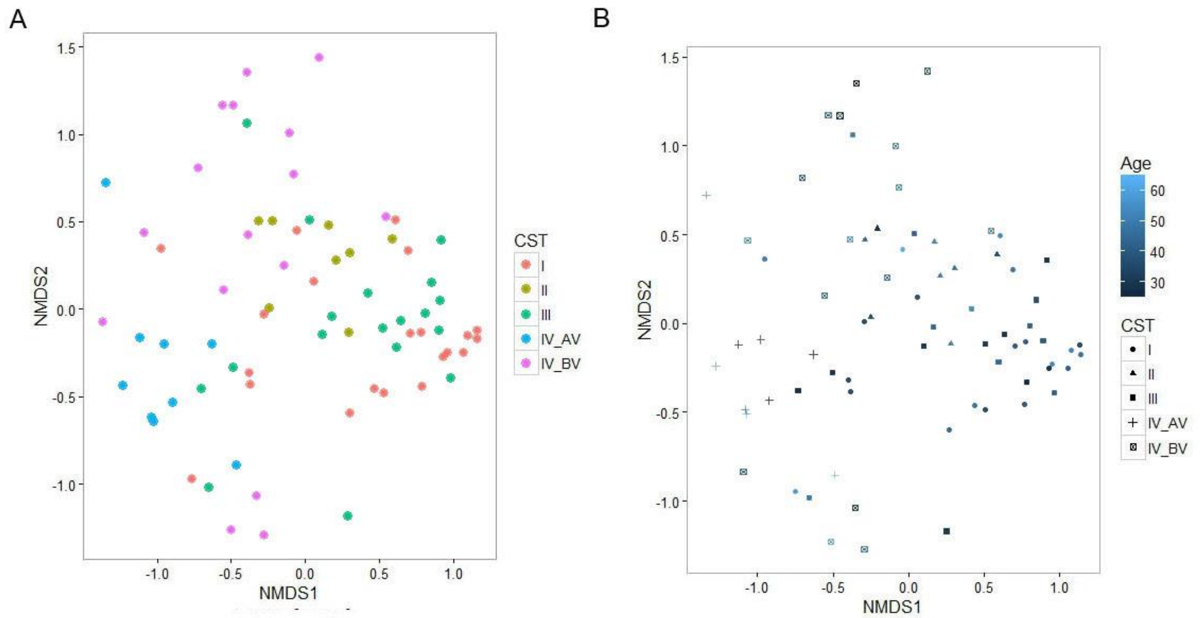

Supplementary Fig. S3. Beta diversity measure. (A-B) Non-metric Multi Dimensional Scaling (NMDS) plots, based on Bray-Curtis dissimilarities. (A) Samples belonging to different CSTs are indicated with different colour dots. (B) CSTs are indicated with different forms (dots, triangles, cross-marks and squares). Age range are indicated with different blue colour-scales. p=0.001 for CST, p=0.002 for Age and p=0.009 for Age:CST, respectively by PERMANOVA using the adonis() function with 999 permutations.

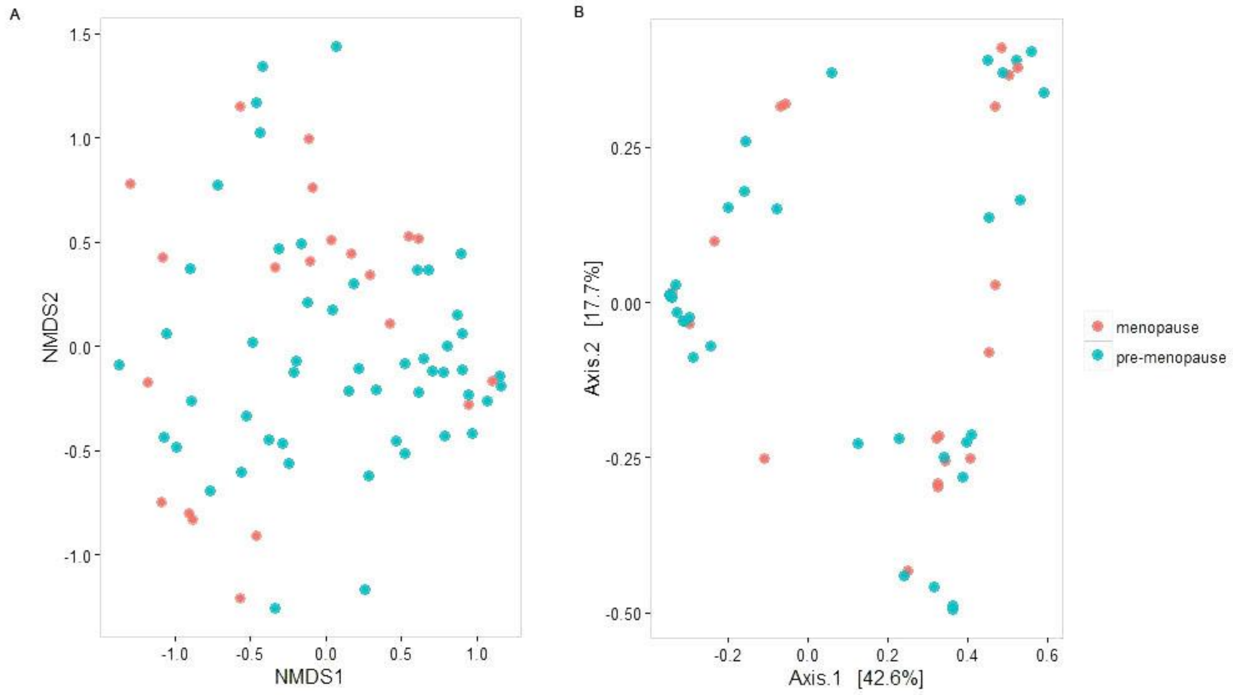

Supplementary Figure S4. **Beta diversity measure.** (A) Non-metric Multi Dimensional Scaling (NMDS) plot and (B) PCoA based on Bray Curtis dissimilarities, correlated with menopause. No statistically significant distribution was obtained by p PERMANOVA using the adonis() function with 999 permutations.
